# Infiltration of myeloid cells into decidua is a critical early event in the labour cascade and post-partum uterine remodelling

**DOI:** 10.1111/jcmm.12012

**Published:** 2013-02-05

**Authors:** Oksana Shynlova, Tamara Nedd-Roderique, Yunqing Li, Anna Dorogin, Tina Nguyen, Stephen J Lye

**Affiliations:** a Samuel Lunenfeld Research Institute, Mount Sinai HospitalToronto, Canada; b Department of Physiology, University of TorontoToronto, Canada; c Department of Obstetrics & Gynecology, University of TorontoToronto, Canada

**Keywords:** decidua, leucocyte infiltration, labour, cytokines, LPS, mifepristone

## Abstract

Leucocyte infiltration in the decidua (maternal–foetal interface) before, during and after term (TL) and preterm labour (PTL) was studied in mouse. We also investigated the mechanism of peripheral leucocyte recruitment into decidua by analysing the tissue cytokine profiles. Decidual tissues were collected during late gestation, TL and post-partum (PP). PTL was initiated on gestational day 15 by intrauterine injection of Lipopolysaccharide (LPS, 125 μg) or progesterone signalling antagonism by RU486. Animals were killed during PTL or PP. Decidua basalis was analysed using FACS and immunohistochemistry. Markers of myeloid cell differentiation (Gr1, Ly6G, Neu7/4, F4/80) were assessed to define tissue monocytes (M), neutrophils (N) and macrophages (Macs). Flow cytometry revealed a significant (*P* < 0.05) increase in decidual Macs prior to TL; M and N numbers increased during TL and further increased during PP, which correlated with immunohistochemistry data. Massive influx of N, but not Macs and M, was detected by FACS during LPS-PTL (*P* < 0.05) but not RU486-PTL. Highest levels of N infiltration into the decidua occurred PP in both LPS and RU486 groups. Decidual infiltration during TL and RU486-PTL was accompanied by an increase in pro-inflammatory cytokines (IL1b and IL6) and CCL2 chemokine; LPS-PTL showed increases in multiple cytokines. PP period following TL and PTL was associated with further up-regulation of multiple cytokines/chemokines (*P* < 0.05). Our data suggest a programme of myeloid cells involvement in parturition with the pre-partum influx of Macs into the decidua contributing to the progression of labour, whereas the later influx of M and N contribute to PP decidual involution.

## Introduction

There is substantial evidence implying that physiological uterine inflammation contributes to the onset of term parturition [1]. Spontaneous labour at term is associated with the infiltration of inflammatory cells into the cervix, myometrium, chorioamniotic membranes and amniotic cavity, even in the absence of infection [2]. Human cervical ripening, an early event in normal labour, is characterized by an accumulation of leucocytes (predominantly macrophages and neutrophils) in the cervical stroma [3]. The human myometrium is infiltrated by immune cells during spontaneous non-complicated labour at term (TL), as well as labour complicated by uterine infection leading to chorioamnionitis [4, 5]. Inflammatory cells also infiltrate the term placenta, maternal decidua and the foetal membranes (where they may contribute to spontaneous membrane rupture) [6]. Pregnant human endometrium (decidua), situated between the foetus and the myometrium, plays an important role in the maintenance of pregnancy and the transition to labour [7]. The decidua is a key intrauterine source of prostaglandins [8, 9] and cytokines [10]. There are numerous reports showing the important role of the human decidua in the first trimester of pregnancy when its high immunologic activity ensures the coordinated paracrine interaction between maternal and foetal compartments [11, 12]. In early pregnancy, the decidua provides a tolerant immune environment supporting trophoblast invasion, vascular remodelling, implantation, placentation and, ultimately, a successful pregnancy [13, 14]. Flow cytometric analysis of human early and term decidual cell suspensions has revealed that almost half of the cells express the leucocyte common antigen (CD45) and are of the haematopoietic lineage [15]. Our previous studies in the rat indicate a large infiltration of macrophage into the myometrium and decidua at term [16, 17]. Decidual macrophage infiltration in the rat immediately before labour preceded that of the myometrium and was 3.8-fold higher [16]. The precise role of the different uterine compartments in the labour process is still debated. We speculate that the premature activation of the maternal immune system, either by infection or by other risk factors, can trigger premature myometrial and/or decidual activation and labour onset causing the delivery of a preterm baby. For instance, macrophage abundance in the decidua of human pregnancies was higher in TL and non-infection associated preterm labour (PTL) than in term non-labouring samples. Neutrophil abundance was unchanged with labour but elevated in PTL with infection [16]. The mechanism of leucocyte infiltration into the pregnant uterus is not fully understood. Immune cells appear to be attracted to the site of inflammation by chemokines (CCL2, CXCL1, *etc*.), and are themselves, a rich source of pro-inflammatory cytokines (IL1β, IL6 and TNFα) and prostaglandins [18, 19]. Data indicate that both uterine compartments (myometrium and decidua [20]) synthesize chemokines that could contribute to the attraction of leucocytes; however, the temporal associations between the synthesis of specific chemokines and the recruitment of leucocytes have not been fully characterized. Importantly, uterine tissues from preterm deliveries (with and without intrauterine infection), show a correlation between cytokine levels and the leucocyte infiltration, suggesting a direct link between the host response to infection and the onset of PTL [21, 22].

We hypothesize that in preparation for labour (term or preterm), peripheral leucocytes are recruited into the maternal–foetal interface (decidua) by locally produced cytokines; they amplify an inflammatory cascade and facilitate labour by activating the decidua and the adjacent myometrium. In this study, we used pregnant murine models to characterize both the decidual tissue cytokine profiles [by real-time PCR (mRNA) and Luminex assays (protein)] and the infiltration of immune cells (monocytes, macrophages, neutrophils, eosinophils) into the decidua [by multi-parameter flow cytometry (FACS) and stereological immunohistochemistry (IHC)]. We examined this relationship during late pregnancy, spontaneous onset of TL and post-partum, as well as during the induction of PTL either by administration of the progesterone antagonist, mifepristone (RU486; a model of non-infection PTL), or by intrauterine infusion of Lipopolysaccharide (LPS, 125 μg; a model of infection-induced PTL). Our results demonstrate coordination between the temporal expression of specific cytokines within the decidua, the influx of leucocytes into this tissue and the induction of physiological and pathological labour. Moreover, they suggest that specific inflammatory mediators and leucocyte subsets may play distinct roles in the onset of labour and post-partum remodelling of uterine tissue.

## Material and Methods

### Animal model

Hsd:ICR (CD-1) outbred mice used for these experiments were purchased from Harlan Laboratories (http://www.harlan.com/). All mice were housed under specific pathogen-free conditions at the Toronto Centre for Phenogenomics (TCP) on a 12L:12D cycle and were administered food and water *ad libitum*. All animal experiments were approved by the TCP Animal Care Committee. Female mice were mated overnight with males and the day of vaginal plug detection was designated gestational day (GD) 0.5 of pregnancy. The average time of delivery was the morning of GD19. Our criteria for labour were based on delivery of at least one pup from average number of 14 in two uterine horns.

### Experimental design

#### Normal pregnancy and term labour

Animals were killed by carbon dioxide inhalation and decidual samples were collected on GD15, GD18, term—not in labour (TNIL), term labour (TL) and 2–6 hrs post-partum (PP). GD15, GD18 tissue was collected at noon; the TNIL sample was collected at 6 a.m. at GD19; the TL sample was collected during active labour once the animals had delivered at least one pup. For each day of gestation, tissue was collected from four to eight different animals.

#### LPS-induced preterm labour

The LPS used for this study was isolated from *E.coli*, serotype 055:B5 (Sigma-Aldrich, St Louis, MO, USA). On GD15 mice underwent mini-laparotomy under general anaesthesia (isofluorane) with intrauterine infusion of 125 μg LPS in 100 μl of sterile saline between two lower amniotic sacs (LPS group) or intrauterine infusion of 100 μl sterile saline (Sham group). Animals (*n* = 4–8 per group) were killed during active LPS-induced PTL or 24 hrs after sham surgery and decidua basalis was collected from both horns. Post-partum samples were collected 2–6 hrs after preterm delivery (LPS PP group).

#### RU486-induced preterm labour

On GD15 of gestation, two groups of mice were injected subcutaneously with either RU486 (150 μg in 100 μl corn oil containing 10% EtOH, 17ß-hydroxy-11ß-[4-dimethylaminophenyl]-17-[1-propynyl]-estra-4,10-dien-3-ne; Mifepristone; Biomol International, Plymouth, PA, USA) or vehicle (100 μl corn oil). Decidual samples were collected from RU486-treated animals during active labour, after delivery of at least one pup (RU486 group), or 24 hrs after injection of the corn oil/ethanol solvent in control mice (Vehicle group) (*n* = 4–8/group). Post-partum samples were collected 2–6 hrs after preterm delivery (RU486 PP group).

### Tissue collection for cytokine expression analysis and immunohistochemistry

For RNA and protein extraction, one uterine horn was placed into ice-cold PBS, bisected longitudinally and dissected away from both pups and placentae. The decidua basalis was cut away from the myometrial tissue, flash frozen in liquid nitrogen and stored at −80°C. The second uterine horn was collected for immunohistochemical analysis: the whole intact uterine horn was cut into 5–10 mm segments and placed in 10% neutral buffered formalin (Harleco, Baltimore, MD, USA) or 4% paraformaldehyde (PFA, Electron Microscopy Sciences, Hartfield, PA, USA) for fixation. Samples were fixed for 24 hrs at 4°C.

### Real-time polymerase chain reaction (PCR) analysis

Total RNA was extracted from the frozen mouse decidua using TRIZOL (Gibco BRL, Burlington, ON, Canada) according to manufacturer's instructions. RNA samples were column purified using RNeasy Mini Kit (Qiagen, Mississauga, ON, Canada), and treated with DNase I (Qiagen) to remove genomic DNA contamination. The process was quality controlled by measuring yield (μg), concentration (μg/l) and 260:280 ratios *via* spectrometry using Nanodrop ND-1000 and sample integrity using Experion system (Bio-Rad, Mississauga, ON, Canada). cDNA synthesis was performed per manufacturer's protocols (iScript cDNA synthesis kit, Bio-Rad). Quantitative real-time PCR was performed using Luminoct SYBR Green QPCR READYMIX (Sigma-Aldrich), CFX-96 Real Time System C1000 Thermal Cycler (Bio-Rad) and a specific pairs of primers (see Table 1). Aliquots (10 ng) of cDNA were used for each PCR reaction run in triplicates. A cycle threshold (Ct) value was recorded for each sample. Each gene was normalized to the expression of three housekeeping genes (*Ppia*, *Tbp* and *Hprt*) by CFX Manager (version 2.1) software and relative expression was calculated for mouse normal gestation using the average of GD15 as the external calibrator in the comparative Ct method (see ABI User Bulletin No. 2). Gene expression for LPS- and RU486-treated animals was presented as the average fold change relative to the Sham or Vehicle.

**Table 1:** Real-time PCR primer sequences of a panel of genes involved in inflammatory response and housekeeping genes

Target genes	Primer sequences	GenBank accession no.
*Il1b*	Forward 5′-GGACCCCAAAAGATGAAGGGCTGC-3′	NM_008361
	Reverse 5′-GCTCTTGTTGATGTGCTGCTGCG-3′	
*Il-6*	Forward 5′-CCTCTCTGCAAGAGACTTCC-3′	NM_031168
	Reverse 5′-CTCCGGACTTGTGAAGTAGG-3′	
*Il-12b*	Forward 5′-AACCAGAAAGGTGCGTTCCTC-3′	NM_008352
	Reverse 5′-ATGCCCACTTGCTGCATGA-3′	
*Tnfa*	Forward 5′-ATGGCCCAGACCCTCACACTCA-3′	NM_013693
	Reverse 5′-TGGTGGTTTGCTACGACGTGGG-3′	
*Csf2*	Forward 5′-TCGAGCAGGGTCTACGGGGC-3′	NM_009969
	Reverse 5′-GTCCGTTTCCGGAGTTGGGGG-3′	
*Ccl2*	Forward 5′-AGG TGT CCC AAA GAA GCT GTA-3′	NM_011333
	Reverse5′-TCT GGA CCC ATT CCT TCT TG-3′	
*Cxcl1*	Forward 5′-CCTGCAGACCATGGCTGGGAT-3′	NM_008176
	Reverse 5′-GTGTGGCTATGACTTCGGTTTGGG-3′	
*Ccl3*	Forward 5′-AGCTGACACCCCGACTGCCT-3′	NM_011337
	Reverse 5′-TCAGGAAAATGACACCTGGCTGGGA-3′	
*Ccl4*	Forward 5′-AGCCAGCTGTGGTATTCCTGACCA-3′	NM_013652
	Reverse 5′-TCATGTACTCAGTGACCCAGGGCT-3′	
*Cxcl2*	Forward 5′-GTTTGCCTTGACCCTGAAGCCCC-3′	NM_009140
	Reverse 5′-CCAGGTCAGTTAGCCTTGCCTTTGT-3′	
*Il-10*	Forward 5′-GCGGCTGAGGCGCTGTCAT-3′	NM_010548
	Reverse 5′-GGCCTTGTAGACACCTTGGTCTTGG-3′	
*Hprt*	Forward 5′-CAGTCCCAGCGTCGTGAT-3′	NM_013556.2
	Reverse 5′-CAAGTCTTTCAGTCCTGTCCATAA-3′	
*Ppia*	Forward 5′-CAC CGT GTT CTT CGA CAT CA-3′	NM_008907.1
	Reverse 5′-CCA GTG CTC AGA GCT CGA AAG-3′	
*Tbp*	Forward 5′-TCCCAAGCGATTTGCTGCAGTCATC-3′	NM_013684
	Reverse 5′-ACTCTTGGCTCCTGTGCACACCA-3′	

### Luminex assay

Frozen decidua samples were crushed under liquid nitrogen and homogenized in bicine lysis buffer [25 mM Bicine, 150 mM NaCl, pH 7.6] supplemented with 100 μM sodium orthovanadate and protease inhibitor cocktail tablets (CompleteTM Mini; Roche, Quebec, Canada). Samples were spun at 12,000 g for 15 min. at 4°C, the supernatant was transferred to a fresh tube to obtain a crude protein lysate and stored at −20°C until assayed. Total protein concentrations were determined using Bio-Rad assay (Bio-Rad). A quantity of 250 μg of protein from tissue homogenates of each decidual sample were used for multiplex assay. Tissue cytokine levels were quantified using Bio-Plex Pro Mouse Cytokine 7-Plex Array kit (Bio-Rad). Multiplex assay was performed on Luminex 200 system and Bioplex HTF (Bio-Rad) in accordance with the manufacturer's instructions. Standards and each sample were analysed in duplicate. Data analysis was performed using Bio-Plex Manager, version 5.0 (Bio-Rad) and presented as concentrations (pg/ml).

### Tissue dispersion for flow cytometry

Uteri were dissected, the decidua was separated from the myometrium; embryos and yolk sacs were discarded. Decidua basalis were finely minced with scissors and enzymatically dissociated in HBSS buffer with 10% FBS, containing Collagenase I (1 mg/ml, Sigma-Aldrich), DNAse (0.15 mg/ml, Roche) and Hyaluronidase, type 1S (200 μ/ml, Sigma-Aldrich) for 2 hrs at 37°C with agitation. The tissues were manually pipetted every 30 min. to improve dispersion (repeated four times) prior to filtration. After incubation, the single-cell suspensions were passed three times through a syringe with an 18-gauge needle, followed by three passes through a 23-gauge needle, filtered through a 40-μm cell strainer, and pelleted by centrifugation (500 × *g*, for 5 min. at 4°C).

### FACS analysis

Staining procedure was performed as described earlier [23] with some modifications. Briefly, dispersed cells were pre-treated with FcγR-blocking mAb 2.4G2 (BD Biosciences, San Diego, CA, USA) together with a combination of up to six directly conjugated fluorescent Abs and Fixable Dead Cell Green Stain Kit (Invitrogen, Eugene, OR, USA). Cells were incubated for 60 min. in the dark on ice. RBC lysis and cellular fixation was completed using BD FACS lysing solution (BD Biosciences). Cells were washed and samples were run within 24 hrs on a 2-laser 7-colour BD Biosciences FACS Aria flow cytometer using BD FACS Diva software (BD Biosciences). After the initial gating on forward-*versus*-side scatter plots, uterine cell populations were gated on all viable leucocytes and APC-Cy7-conjugated anti-CD45 antibody (clone30-F11; BD Biosciences). The cells were then delineated into subsets with antibodies against F4/80 (BM8, C1:A3-1), neutrophils (Neu7/4), Ly6C/G (Gr-1, RB6-8C5) and Siglec F purchased from BD Biosciences and AbD Serotec and variously conjugated to FITC, RPE-Alexa Fluor 647, PerCP-Cy5.5, PE, Alexa Fluor 647, PE-Cy7. Isotype controls were used to assess background fluorescence; FMO controls were used to verify subset gates.

### Immunohistochemistry

The formalin-fixed uterine tissues were gradually dehydrated in ethanol and embedded in paraffin. Sections of 5 μm thickness were collected on Superfrost Plus slides (Fisher Scientific, Nepean, ON, Canada). Paraffin sections were deparaffinized and rehydrated. After immersion in 3% hydrogen peroxide (Fisher Scientific, Fair Lawn, NJ, USA), the antigens were unmasked using a microwave heating retrieval treatment in 10 mM sodium citrate solution (pH6) for formalin-fixed tissue and using 0.125% trypsin for PFA-fixed tissue. Blocking was performed for 1 hr with DAKO Protein Serum-Free Blocking solution (DAKO Corporation, Carpinteria, CA, USA). Formalin-fixed tissue was incubated with primary anti-Neu7/4 monoclonal rat antibody (1:100; Cedarlane, Burlington, ON, Canada) overnight. According to the manufacturer's protocol, Neu7/4 recognizes the polymorphic 40 kD antigen expressed by polymorphonuclear cells, but absent on resident tissue macrophages. Our current study and earlier reported flow cytometry data [24], however, indicate that Neu7/4 can also recognize monocytes. PFA-fixed tissue was incubated with primary anti-F4/80 monoclonal rat antibody (1:100; BioLegend, San Diego, CA, USA). F4/80 recognizes the 160 kD glycoprotein expressed by murine macrophages and has low expression on monocytes and eosinophils. For the negative controls, ChromPure non-specific rat IgGs (Jackson ImmunoResearch Laboratories, West Grove, PA, USA) were used at the same concentration and sections were also incubated with secondary antibodies in the absence of primary antibodies. Detection was accomplished using biotinylated rabbit anti-rat IgG (1:200, Vector Laboratories, Burlingame, CA, USA) in combination with Streptavidin HRP (DAKO Corporation). Final visualization was achieved using Dako Liquid DAB+ Substrate Chromogen System (DAKO Corporation). Counterstaining with Harris' Hematoxylin (Sigma-Aldrich) was carried out before slides were mounted with Cytoseal XYL (Thermo Scientific, Kalamazoo, MI, USA).

### Assessment of leucocyte infiltration using NewCast software

Infiltration of macrophages and neutrophils was quantified using NewCast stereology software with systematic randomized sampling of 5% of the total decidual area. Uterine tissues from different gestational days, TL, from LPS PTL and RU486 PTL groups along with appropriate controls were observed on an Olympus BX61 microscope and recorded using an Olympus DP72 camera. The population of leucocytes was assessed for Neu7/4 and F4/80 immunostaining to identify tissue neutrophils/monocytes and macrophages respectively. In each uterine tissue sample, NewCast Software, part of Visiopharm integrator system 3.6.5.0, was used to generate 30–40 non-contiguous, randomly selected fields of myometrium. Cells positive for Neu7/4 and F4/80 and total nuclei in each field were counted at 20× magnification. The number of cells having positive staining was divided by total number of cells in the field and multiplied by 100 to determine percentages of Neu7/4 or F4/80 positive cells.

### Statistical analysis

Grubbs outlier test was utilized to identify and exclude outliers from all data sets. To determine differences between groups, we subjected gestational profiles, LPS and RU486 profiles to a one-way analysis of variance (anova) followed by Newman–Keuls post-test (for normally distributed data) or to Kruskal–Wallis non-parametric test followed by Dunns post-test (for non-normally distributed data). Normality test and equal variance test were performed by Sigma Stat statistical program (version 3.11) and where required, the data were transformed by natural logarithm function to obtain a normal distribution. Statistical analysis was carried out using Sigma Stat with the level of significance set at *P* < 0.05.

## Results

### Leucocyte infiltration throughout late gestation, term labour and post-partum in the mouse decidua

**Fig. 1:**
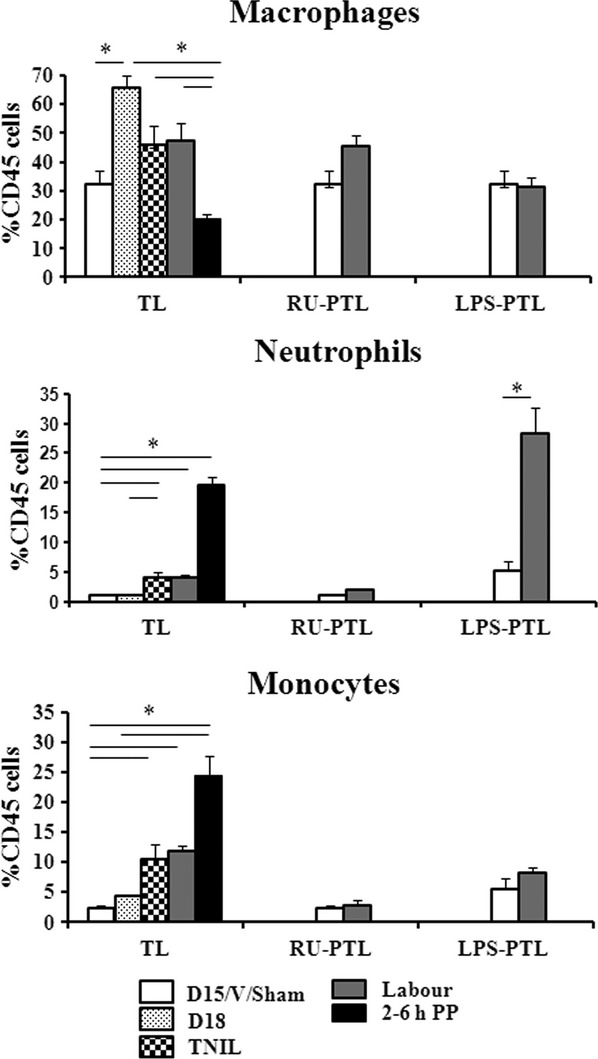
Recruitment of myeloid cells in the mouse decidua. Decidual suspensions were stained with anti-CD45, F4/80, Gr1 and Neu7/4. Leucocytes were gated on CD45 and further analysed on the Neu7/4 *versus* Gr1 dot plot. Macrophages were defined as F4/80^++^ or CD11b^++^ and Neu7/4^−^. Monocytes were identified as Neu7/4^++^ and Gr1^−^ to Gr1^+/−^. Neutrophils were defined as Neu7/4^+^, Gr1^++^ [23]. Decidua was analysed during late gestation, term not in labour, term labour and 2–6 hrs post-partum (TL), LPS-induced PTL (LPS PTL) and RU486-induced PTL (RU PTL). Shown are non-labouring samples (GD15, LPS Sham surgery or RU Vehicle, white bars), GD18 (term not in labour, doted bars), GD19 (term not in labour, checkered bars), term and preterm labouring samples (grey bars) or post-partum samples (black bars). Data represent mean ± SEM of four to eight decidual samples. Significant difference with GD15/RU486 Vehicle/Sham LPS is indicated by *P < 0.05.

We analysed the infiltration of myeloid immune cells into mouse decidua by flow cytometry throughout different phases of pregnancy: late gestation (GD15 and GD18), before labour (GD19, TNIL), during active TL and early PP (Fig. 1). Decidual cell suspensions were stained with anti-CD45, anti-F4/80, anti-Siglec-F, anti-Gr1 and anti-Neu7/4 as described in [23]. Live leucocytes were gated on CD45 and further analysed on the Neu7/4 *versus* F4/80 dot plot (Figure S1). Neu7/4^++^ cells were further analysed on the Neu7/4 *versus* Gr1 plot. Monocytes were identified as Neu7/4^++^ and Gr1^low^ to Gr1^+/−^ cells. Neutrophils were defined as Neu7/4^+^, Gr1^++^ population. Macrophages were defined as F4/80^++^, Neu7/4^low^ cells, they were also CD11b^++^. Population of cells defined as F4/80^+^, Neu7/4^low^ Ly6G^−^ strongly expressed the eosinophil-specific marker, Siglec-F [23].

**Fig. 2:**
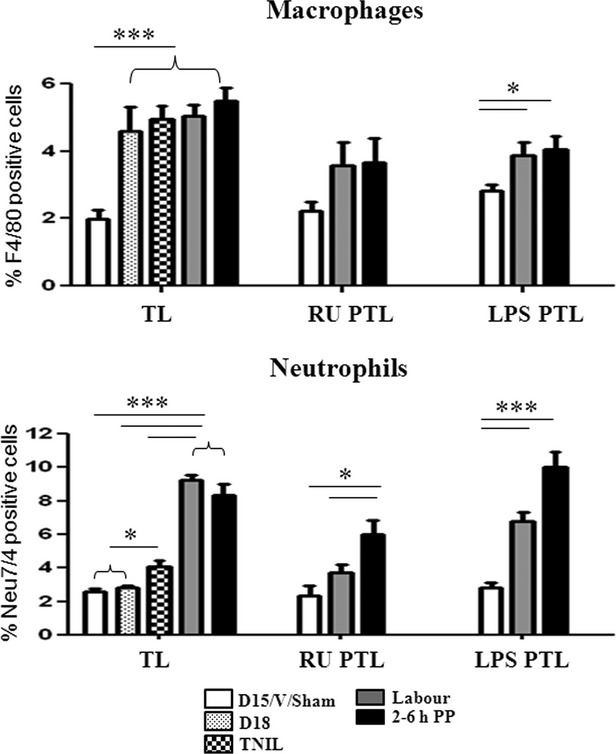
Neutrophil and macrophage infiltration into the mouse decidua during TL and PTL. Neutrophils were identified using anti-Neu7/4 antibody and macrophages were identified using anti-F4/80 antibody. NewCast software was used to quantify neutrophil and macrophage numbers relative to total cell numbers in the decidua during late gestation, term labour and post-partum (TL), LPS-induced PTL and post-partum (LPS PTL) and RU486-induced PTL and post-partum (RU PTL). Shown are non-labouring samples (GD15, LPS Sham or RU Vehicle, white bars), GD18 (doted bars), GD19 (term not in labour, checkered bars), term and preterm labouring samples (grey bars) or post-partum samples (black bars). Results were expressed as mean ± SEM (*n* = 4–6). Significant difference with GD15/RU486 Vehicle/Sham LPS is indicated by *P < 0.05 and **P < 0.01.

Macrophages were the largest population of immune cells present in pregnant mouse decidua, representing 33.5% of all leucocytes (Fig. 1). They doubled in number by GD18 (term) as compared with GD15, were elevated before (TNIL) and during spontaneous labour (TL) and were present in decidua after birth (Figs 1 and 2, *P* < 0.05). The number of decidual monocytes (Neu7/4^++^, Gr^+/−^) detected by flow cytometry significantly increased at term (TNIL) as compared with GD15 (10.5% *versus* 2.4%), were high during labour (11.8%) and were dramatically up-regulated through the early PP period (24.3%, *P* < 0.01, Fig. 1, Figure S1A). Neutrophils (Neu7/4^+^, Gr^++^) were present in decidua throughout late gestation, they increased slightly before and during labour as compared with GD15 (4.1% *versus* 1.1%) and were significantly up-regulated PP (19.7%, *P* < 0.01, Fig. 1). At term and during labour decidual macrophages were dominating population of myeloid cells, however, after parturition majority of immune cells in decidua were newly infiltrated monocytes and neutrophils. Tissue eosinophils were low (around 1%) and unchanged during gestation, TL and early PP (data not shown).

NewCast software was used to confirm the flow cytometry data by quantifying neutrophils and macrophages immunostained *in situ*. Similar to FACS, immunohistochemical analysis indicates that F4/80 positive macrophages were significantly increased on GD18 (4.6 ± 0.7%) as compared with GD 15 (2.0 ± 0.3%), were elevated during TL (5.1 ± 0.3%) and early PP (5.5 ± 0.3%, *P* < 0.05, Fig. 2 and Figure S2). The number of neutrophils (defined as cells stained positively for Neu7/4) was low during late gestation (2.6 ± 0.2% of all cells), but significantly increased in decidua during TL (9.3 ± 0.3%) and PP period (8.4 ± 0.5%, *P* < 0.05, Fig. 2 and Figure S2). We assume that some of the Neu7/4 positive cells were actually monocytes as they can express this marker. It was previously reported that Neu7/4 can recognize monocytes along with neutrophils and that F4/80 has low expression on monocytes [24].

### Decidual activation during preterm labour

Preterm labour was induced (1) by artificial progesterone blockade using mifepristone (non-infectious, sterile PTB [25]) or (2) by intrauterine injection of 125 μg LPS (the well-known model of infectious PTB [26]) on GD15 pregnant mice. Both treatments resulted in PTL within 24 hrs with no maternal mortality. Flow cytometry was used to compare the inflammatory cell populations in decidua basalis of two PTL models *versus* TL (Fig. 1, Figure S1B). Analysis of decidual cell suspensions from LPS-induced preterm labouring mice showed a dramatic increase in tissue neutrophils as compare with the decidua from the Sham group (5.1% *versus* 28.2%, *P* < 0.01). During RU486-induced PTL, the number of macrophages was unchanged and no infiltration of myeloid immune cells (N and M) into the decidua was detected. No significant change in eosinophils was observed with any of the treatments.

Recruitment of macrophages and neutrophils into the mouse decidua was also assessed *in situ* in both models of PTL. In accordance with flow cytometry results, no significant change in immunopositive macrophages or neutrophils/monocytes was detected in the decidua during RU486-induced PTL (Fig. 2, Figure S2C). In contrast, the LPS-PTL group showed a significant increase in the number of neutrophils and macrophages in decidua as compared with the Sham group. Importantly, in the PP period, the number of neutrophils/monocytes increased significantly following both LPS and RU486-induced PTL (*P* < 0.05, Fig. 2, Figure S2D).

### Decidual cytokine levels at late gestation, during term labour and post-partum

**Fig. 3:**
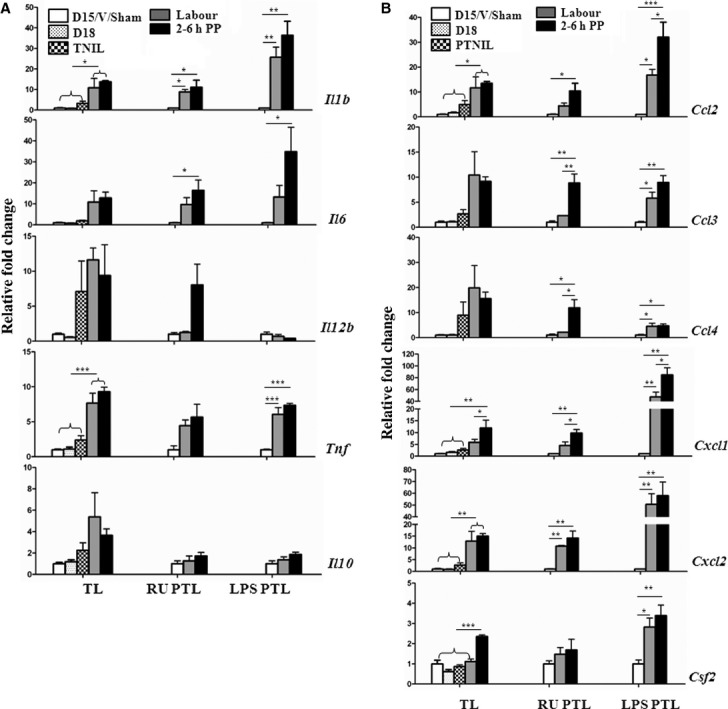
Changes in cytokine mRNA levels in the mouse decidua during normal gestation, term labour and post-partum (TL group), LPS-induced PTL and post-partum (LPS PTL group) and RU486-induced PTL and post-partum (RU PTL group). (**A**) Pro-inflammatory (*Il1b, Il6, Il12b, Tnf*) and anti-inflammatory (*Il10*) cytokines; (**B**) Chemokines *Ccl2 (Mcp1), Ccl3 (Mip1a), Ccl4 (Mip1b), Csf2 (Gmscf), Cxcl1 (KC or Groa)* and *Cxcl2 (Mip2a)* mRNA expression were detected by Real-Time RT-PCR. Shown are samples collected from late pregnant mice (GD15, RU Vehicle or LPS Sham, white bars), GD18 (doted bars), GD19 (term not in labour, checkered bars), term and preterm labouring samples (grey bars) or decidual samples collected 2–6 hrs post-partum (black bars). Results were expressed as mean ± SEM (*n* = 4). Significant difference with GD15/RU486 Vehicle/Sham LPS is indicated by *P < 0.05 and **P < 0.01.

**Fig. 4:**
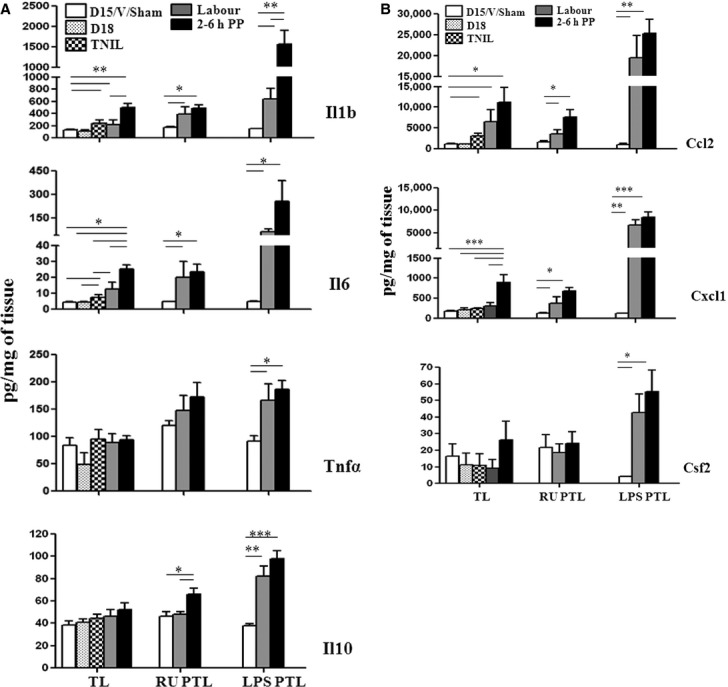
Changes in cytokine protein levels in the mouse decidua during normal gestation, term labour and post-partum (TL group), LPS-induced PTL and post-partum (LPS PTL group) and RU486-induced PTL and post-partum (RU PTL group). (**A**) Pro-inflammatory (IL1b, IL6, TNFα) and anti-inflammatory (IL10) cytokines; (**B**) Chemokines CCL2, CXCL1 and CSF2 protein expression were detected by multiplex magnetic bead assay. Shown are decidual samples from non-labouring mice (GD15, RU Vehicle or LPS Sham, white bars), GD18 (doted bars), GD19 (term not in labour, checkered bars), term and preterm labouring samples (grey bars) or decidual samples collected 2–6 hrs post-partum (black bars). Results were expressed as mean ± SEM (*n* = 4–8). Significant difference with GD15/RU486 Vehicle/Sham LPS is indicated by *P < 0.05 and **P < 0.01.

The expression of multiple pro- and anti-inflammatory genes were evaluated by Real-Time PCR during late gestation, TL, and PP. Pro-inflammatory cytokines tumour necrosis factor-α (*Tnf*), interleukin (Il)1β *(Il1b)*, *Il6* and *Il12* as well as two major neutrophil chemoattractants (C-X-C motif) ligand 1 (*Cxcl1,* also known as Neutrophil-activating protein 3 (NAP-3) or KC), *Cxcl2* (also known as macrophage inflammatory protein 2-alpha, Mip2a) and monocyte chemoattractant (C-C motif) Ligand 2 (*Ccl2,* also known as monocyte chemoattractant protein-1, Mcp1) mRNA levels all were significantly up-regulated during labour and PP (Fig. 3, Table 2). *Ccl4* (also known as Macrophage inflammatory protein 1 beta, Mip1b), *Ccl3* (also known as Macrophage inflammatory protein 1 alpha, Mip1a) and anti-inflammatory cytokine *Il10* genes were also increased during TL but significance was not reached due to a high variability between samples (Fig. 3A). Transcript levels of growth factor *Csf2* (granulocyte–macrophage colony-stimulating factor) regulating neutrophil/monocyte differentiation increased in decidua PP 2–6 hrs after parturition (Fig. 3B). The expression of corresponding cytokine proteins partially confirmed changes in cytokine transcripts. Using multiplex protein assay we detected a significant increase in decidual IL1b and IL6 pro-inflammatory cytokines and Ccl2 chemokine during TL and PP (*P* < 0.05, Fig. 4, Table 3). CXCL1 protein was only increased PP (*P* < 0.05) whereas no change in the protein level of TNFα, IL10 and CSF2 was observed (Fig. 4, Table 3).

**Table 2:** Changes in cytokine mRNA levels in the mouse decidua during normal gestation (GD15), late gestation (GD18), term not in labour GD19 (TNIL), term labour (TL) and 2–6 hrs post-partum (PP), RU486-induced preterm labour (RU-PTL), Vehicle-treated GD15 (Vehicle) and post-partum (RU-PP), LPS-induced preterm labour (LPS-PTL), sham operated GD15 (Sham) and post-partum (LPS-PP)

Cytokines	*Il1b*	*Il6*	*Il10*	*Il12*	*Tnfa*	*Ccl2*	*Ccl3*	*Ccl4*	*Cxcl1*	*Cxcl2*	*Csf2*
GD15	1.0 ± 0.3†,‡	1.0 ± 0.4†,‡	1.0 ± 0.3	1.0 ± 0.2‡	1.0 ± 0.2†,‡	1.0 ± 0.2†,‡	1.0 ± 0.4	1.0 ± 0.3	1.0 ± 0.2†	1.0 ± 0.5†,‡	1.0 ± 0.4†
GD18	0.9 ± 0.3†,‡	0.7 ± 0.3†,‡	1.2 ± 0.4	0.5 ± 0.2‡	1.1 ± 0.6†,‡	1.7 ± 0.6†,‡	1.1 ± 0.3	1.0 ± 0.3	1.6 ± 0.7†	0.9 ± 0.3†,‡	0.6 ± 0.2†
TNIL	3.3 ± 2.1†,‡	3.8 ± 3.8†,‡	2.3 ± 1.4	7.1 ± 8.8	2.4 ± 1.3†,‡	4.9 ± 3.2†,‡	2.7 ± 1.7	8.9 ± 10.6	2.6 ± 1.3†	2.7 ± 2.0†,‡	0.9 ± 0.2†
TL	10.8 ± 9.3	10.9 ± 10.8	5.4 ± 4.5	11.6 ± 3	7.7 ± 2.8	11.7 ± 8.8	10.4 ± 9.3	19.9 ± 18	5.9 ± 2.5†	12.9 ± 8.4	1.1 ± 0.2†
2-6 h PP	16.3 ± 5.1	12.8 ± 5.7	3.7 ± 1.2	9.4 ± 8.8	9.3 ± 1.3	13.6 ± 1.4	9.2 ± 1.8	15.6 ± 5.2	11.9 ± 6.7‡	15.0 ± 2.1	2.4 ± 0.2
Vehicle	1.0 ± 0.3	1.0 ± 0.4	1.0 ± 0.5	1.0 ± 0.5†	1.0 ± 1.1	1.0 ± 0.5	1.0 ± 0.6	1.0 ± 0.9	1.0 ± 0.2	1.0 ± 0.3	1.0 ± 0.3
RU-PTL	8.8 ± 2.3*	9.7 ± 5.6*	1.1 ± 0.8	2.8 ± 3.1†	4.4 ± 1.7*	4.4 ± 1.9*	3.6 ± 2.6*	2.9 ± 1.5*	4.5 ± 3.1*	9.0 ± 3.5*	1.5 ± 0.7
RU-PP	11.0 ± 7.1*	16.4 ± 9.8*	1.7 ± 0.7	8.1 ± 5.9	5.7 ± 3.7*	10.4 ± 6.2*	8.8 ± 3.6*	12 ± 6.6*	9.8 ± 3.0*	14.2 ± 5.9*	1.7 ± 1.1
Sham	1.0 ± 0.2	1.0 ± 0.5	1.0 ± 0.6	1.0 ± 0.6	1.0 ± 0.2	1.0 ± 0.2	1.0 ± 0.3	1.0 ± 0.5	1.0 ± 0.1	1.0 ± 0.1	1.0 ± 0.4
LPS-PTL	25.7 ± 9.7*	52.5 ± 68.2*	1.4 ± 0.5	0.7 ± 0.5	6.1 ± 2.0*	16.8 ± 4.7*,†	5.8 ± 2.5*	4.5 ± 2.5*	47.3 ± 16.7*	50.5 ± 18.2*	2.8 ± 0.9*
LPS-PP	36.4 ± 13.5*	34.8 ± 23.3*	1.9 ± 0.4	0.4 ± 0.1	7.4 ± 0.6*	32.1 ± 12*	8.9 ± 2.8*	4.7 ± 1.5*	84.5 ± 25*,†	57.9 ± 23.1*	3.4 ± 1.0*

Results were expressed as average ± SD (*n* = 4–8).

* Different from GD15/Vehicle/Sham (*P* < 0.05);

† different from PP (*P* < 0.05);

‡ different from TL (*P* < 0.05).

**Table 3:** Changes in cytokine protein levels (pg/ml) in the mouse decidua during normal gestation (GD15), late gestation (GD18), term not in labour GD19 (TNIL), term labour (TL) and 2–6 hrs post-partum (PP), RU486-induced preterm labour (RU-PTL), Vehicle-treated GD15 (Vehicle) and post-partum (RU-PP), LPS-induced preterm labour (LPS-PTL), sham operated GD15 (Sham) and post-partum (LPS-PP)

Cytokines	Il1b	Il6	Il10	Tnfa	Cxcl1	Ccl2	Csf2
GD15	136.9 ± 14.4†,‡	4.5 ± 1.0†,§	38.3 ± 7.5	83.7 ± 28.7	176.5 ± 44.6†	1127 ± 307†,‡,§	16.0 ± 15.1
GD18	114.1 ± 31.4†,‡	4.5 ± 1.0†,§	41.1 ± 5.9	49.0 ± 41.7	210 ± 96.9†	1149 ± 131†,‡,§	11.1 ± 14.0
TNIL	236.2 ± 109.4*,†	7.4 ± 3.3†	44.2 ± 7.7	95.4 ± 34.6	238 ± 59.6†	3030 ± 1416†,§	9.2 ± 10.0
TL	223.3 ± 134.0*,†	12.5 ± 9.4*,†	46.3 ± 9.9	88.8 ± 32.3	312.4 ± 160†	6580 ± 5789	9.2 ± 10.0
2-6 h PP	500.2 ± 127.1*,†	25.0 ± 5.4	52.3 ± 11.9	93.6 ± 16.0	902 ± 371.8	11212 ± 7269	26.4 ± 22.0
Vehicle	175 ± 19.4	4.7 ± 0.7	46.5 ± 8.2†	120.3 ± 16.6	126.8 ± 27.9	1727 ± 497	21.6 ± 16.0
RU-PTL	390 ± 231.5*	20.0 ± 19.0*	47.8 ± 5.1†	147.4 ± 54.6	380.2 ± 326.2*	3520 ± 2152*,†	18.7 ± 10.0
RU-PP	485.4 ± 118.7*	23.3 ± 10.0*	65.9 ± 10.9	172.9 ± 52.5	687.9 ± 153*	7576 ± 3555*	21.3 ± 14.0
Sham	151.2 ± 14.0	4.7 ± 0.7	38.1 ± 3.6	92.0 ± 19.3	132.3 ± 13	1076 ± 487	4.2 ± 1
LPS-PTL	639.9 ± 338.3*	148.5 ± 25.1*,†	82.2 ± 17.9*	166 ± 60*	6741 ± 2347*	19562 ± 10673*	42.6 ± 23.0*
LPS-PP	1566.2 ± 679.1*	256.4 ± 263.8*	98.0 ± 14.6*	186 ± 32.6*	8483 ± 2367*	25255 ± 6895*	55.4 ± 26.0*

Results were expressed as average ± SD (*n* = 4–8).

* Different from GD15/Vehicle/Sham (*P* < 0.05);

† different from PP (*P* < 0.05);

‡ different from TNIL (*P* < 0.05);

§ different from TL (*P* < 0.05).

### Cytokine expression in decidua during PTL

The expression of cytokine and chemokine genes known to be involved in macrophage and neutrophil recruitment into the gestational tissues was studied in decidual samples collected during both preterm mouse models during PTL and 2–6 hrs PP. We noticed a similarity between cytokine profile discovered in decidua collected from term labouring mice and RU486-induced PTL. Comparable to our TL model *Il1b, Il6, Tnfa* and *Cxcl2* mRNA levels were significantly up-regulated during non-infectious RU486-induced PTL and further increased in the PP period. Importantly, *Il12, Ccl2, Ccl3, Ccl4* and *Cxcl1* transcripts were also significantly elevated PP as compared with Vehicle and PTL (Fig. 3A and B, Table 2). In accordance with the transcript levels, a significant increase in protein levels of pro-inflammatory cytokine IL1b and IL6 as well as chemokines CCL2 and CXCL1 was detected by Bioplex assay in decidua during and after RU486-induced PTL as compared with Vehicle sample. Anti-inflammatory cytokine Il10 was up-regulated PP (*P* < 0.05, Fig. 4A, Table 3), however, there was no change in CSF2 and TNFα proteins.

In contrast with TL and RU486-induced PTL, the gene and protein profile of LPS-induced PTL in mice shows a robust induction of majority of pro-inflammatory cytokines and chemokines we studied. *Il1b*, *Il6*, *Tnf*, *Ccl2, Ccl3, Ccl4, Cxcl1, Cxcl2, Csf2* decidual transcript levels all were up-regulated during LPS-induced PTL (*P* < 0.05, Fig. 3, Table 2). In agreement with transcript levels pro-inflammatory IL1b, IL6, TNFα, as well as anti-inflammatory IL10 protein concentrations were significantly increased in the mouse decidua during preterm parturition compared with Sham samples; all cytokines remained or further increased PP (*P* < 0.05, Fig. 4A, Table 3). In addition, protein expression of chemokines CCL2, CXCL1 and CSF2 was significantly higher in the decidua during LPS-induced PTL compared with Sham and remained elevated PP (*P* < 0.05, Fig. 4B, Table 3).

## Discussion

Only a few studies to date have investigated the inflammatory events in decidual and myometrial tissues prior to, during labour and in PTL with Hamilton *et al*. [16] reporting the presence of elevated decidual macrophages in TL and idiopathic PTL and our group providing a detailed analysis of the myeloid cell infiltration into the mouse myometrium [27]. This current study provides a comprehensive characterization of immune cells recruitment into the decidua in normal and preterm labour. Our results demonstrate the presence of localized decidual inflammation in the absence of infections and indicate that this common labour-related physiological phenomenon is a critical element of the labour cascade at term and preterm.

Decidual activation was first proposed more than 20 years ago as an early event in labour [28]. Salafia and colleagues detected decidual inflammation in uterine and placental samples from labouring women with uncomplicated pregnancies [29]. Keski-Nisula *et al*. showed that histological decidual inflammation (in the absence of infection) was rare before the onset of labour (6%), increased significantly during labour before membrane rupture (29% of all cases) and was correlated with cervical dilatation [4]. Spontaneous labour at term was reported to be associated with changes in the distribution and proportion of decidual leucocyte subpopulations in uncomplicated pregnancies [30]. In contrast with these results, Osman and colleagues reported no significant difference in the density of inflammatory cells (leucocytes, macrophages, neutrophils, T lymphocytes and B lymphocytes) in amnion and choriodecidua after labour compared with before labour [2]. They have been suggested that the myometrium and foetal membranes play complementary roles during the process of labour—the trigger to parturition being delivered from the foetal membranes (possibly through signals received from the foetus[31]) with subsequent leucocyte invasion stimulated within the myometrium to sustain and amplify the process of parturition *via* PG production [32, 33]. The current study was undertaken to examine the possibility that the factor(s), produced by decidua, can activate the inflammatory response causing the influx of leucocytes in uterine tissues, leading to the reinforcement of myometrial contractile activity.

Accumulating evidence supports the concept that labour is associated with the development of physiological inflammation in different uterine compartments. In agreement with previous studies, we found macrophages to be the most abundant leucocyte subpopulation in the decidua [2, 16]. Macrophages comprise about 25% of CD45+ leucocytes in the pregnant human uterus and this relative percentage remains fairly stable throughout gestation [15, 34]. Our flow cytometry data show that in mouse decidua around 30% of immune cells on GD15 were macrophages and their number doubled on GD18 (24 hrs prior to TL) indicating their potential role in labour initiation. Large numbers of CD11b-positive macrophages (around 50% of all CD45+ cells) were present in mouse decidua immediately before (TNIL) and during TL, in addition to the significantly increased infiltration of monocytes. CD11b antigen (the b2 integrin, complement type 3 receptor) is a common leucocyte activation marker. According to our flow cytometry analysis, a majority of decidual tissue macrophages, monocytes and neutrophils expressed this marker.

Consistent with these data on mouse decidual immune cells profiles, our group and others have observed a peak in myometrial [27] and cervical [35] macrophage number around GD18 in mice, with a decline to non-pregnant macrophage levels by day 1PP. The timing of macrophage increase in the pregnant mouse uterus before TL suggests that these cells could play a role in the labour onset and may be required for the rapid infiltration and function of other subtypes of immune cells (possibly neutrophils, monocytes or T cells) during the TL and PP involution process. We have reported earlier that there was apparent macrophage infiltration within the rat myometrium, enhanced along the myometrium–decidua junction and in decidual stroma [17]. The current data indicate a similar infiltration in the mouse myometrial–decidual border. We speculate therefore that decidual macrophages could migrate to the adjacent myometrium where they participate in the activation of uterine muscle and later in the process of PP uterine involution. It is equally possible that decidual leucocytes may activate the adjacent myometrium and/or amniochorion *via* paracrine actions resulting in the increase of cytokines, MMPs and prostaglandins, leading to myometrial activation and foetal membrane rupture (rev in [16]). In addition, flow cytometry of mouse decidua recorded a 10-fold increase (2.4% *versus* 24.1%) in monocytes and 18-fold increase in neutrophils (1.1% *versus* 19.7%) during early PP as compared with GD15. The relative percentage of macrophages decreased after birth due to the dramatic increase in the amount of newly infiltrated monocytes and neutrophils; however, the absolute number was unchanged as shown by our *in situ* analysis. These activated decidual macrophages could be the source of pro-inflammatory cytokines, IL1b and IL6, detected concomitantly in mouse decidua. During TL protein expression of IL1b was 10 times higher in decidua than in the myometrium from the same animals (Fig. 4 and [27]). Cytokines, particularly IL1b and TNFα, can prime uterine myocytes for contraction and labour *via* increased PGHS2 expression and enhanced prostaglandin production in the amnion, chorion, decidua and myometrium [19, 36].

In agreement with observations from human decidual samples [16], neutrophil numbers in mouse decidua were low during TL in the absence of infection. This is also consistent with our previous results showing that neutrophil numbers do not increase in the mouse myometrium [27] until after birth, and with similar reports on the mouse cervix [23, 35]. These data suggest a role for neutrophils in PP uterine remodelling rather than in the initiation of cervical ripening or myometrial activation at parturition. The similarity in the timing of immune cells appearance in two adjacent uterine compartments indicates that the cells could have originated from the same source and/or are regulated by the same biochemical signal.

Next, we addressed the mechanisms regulating immune cell infiltration into the decidua. It was shown that human choriodecidua but not amnion was responsible for the enhanced chemotactic activity through expression of CCL2, CXCL10 (monocytes/macrophages attractants) and CXCL8 and CCL3 (neutrophil recruiter/activators) [37, 38]. Interestingly, monocyte chemotactic activity induced by human foetal membrane extracts was 36-fold higher in women after TL as compared with women with no labour, accompanied by 3-fold increase in neutrophil chemotaxis [37]. We reported recently that human [1] and rodent [17, 27] myometrium could also participate in the physiological uterine inflammation by the synthesis and release of multiple pro-inflammatory mediators. Our current studies, however, demonstrate that mouse decidua provides much stronger chemotactic stimulus than the myometrium for macrophage/monocyte infiltration before and during parturition. For example on GD15, [CCL2] was 1127 ± 153 pg/ml/mg of tissue in decidua, which is seven times higher than in the adjacent myometrium (150 pg/ml/mg) [27], with similar levels detected at GD18 (1149 ± 65 pg/ml). Moreover, during term parturition [CCL2] was 6580 ± 2892 pg/ml which is 22 times higher than in the myometrium (296 ± 44 pg/ml/mg) and [CXCL1] in mouse decidua was 312 pg/ml/mg which is five times higher than in the myometrium (60 pg/ml/mg) from the same animal cohort [27]. Surprisingly, however, none of the cytokines we studied in mouse decidua or myometrium [27] was increased on GD18, which left open the question of the putative signal triggering macrophage migration into either of these uterine compartments. We have potential explanations for this phenomenon. Firstly, multiple other chemokines involved in leucocyte extravasation and activation exist (*i.e*. CSF1/MCSF, CCL5/RANTES), which could provide the stimulus for macrophage migration. Secondly, it is possible that hormonal microenvironment regulates macrophage recruitment into the decidua, specifically the increase in oestrogen/progesterone ratio: it was shown that the administration of oestrogen and/or progesterone to ovariectomized mice restore recruitment of macrophages into the uterus [39]. Thirdly, decidual tissue itself can locally stimulate proliferation of resident macrophages [40, 41]. The origin of the decidual (and myometrial) macrophages has been unclear. It was suggested recently that resident macrophages in the liver, brain, spleen, kidney, pancreas and lung are derived from the yolk sac originated precursors, the myeloid lineage that is distinct from haematopoietic stem cells [42]. These macrophages persist, at least in part, in adult mice and likely self-renew within their respective tissues of residence, independently of bone marrow haematopoietic stem cells. This suggests the existence of tissue resident macrophage ‘stem cells’ within those tissues (and likely in the uterus), and opens a new era for the molecular and cellular understanding of myeloid cells responses during acute and chronic inflammation [43].

We found a similar pattern of cytokine expression in mouse decidua during TL and RU486-induced PTL. This is expected, as progesterone withdrawal is fundamental to labour initiation [44, 45]. However, LPS-induced (infectious) PTL was associated with an elevated and accelerated induction of a broader range of cytokines than that seen with TL and RU486-PTL. We also noticed that the inflammatory reactions during PTL were different from TL, with excessive neutrophil infiltration and lack of a surge of macrophages. This may be due to the premature and rapid timing of events, which may not allow sufficient time for infiltration of monocytes and their differentiation to macrophages to occur. Large numbers of resident macrophages, however, were present in the uterus (both myometrium [27] and decidua) on GD15 and their rapid activation by multiple cytokines may contribute to the process of PTL. The current studies on mouse decidua suggest that different mechanisms operate in TL and LPS-PTL, the idea that was proposed earlier by Holt and colleagues for term and preterm cervical ripening in the mouse [46] and supported by our earlier studies on mouse myometrium [27]. In support of different mechanisms operating in TL and LPS-PTL, Gonzalez and colleagues also observed an increase in the expression of genes involved in immunity and inflammation in the mouse cervix of LPS-induced PTL as compared with TL [47]. The expression of a greater number of cytokines at higher levels was maintained in decidua throughout the PP period. Importantly, the expression of cytokines increased also in the mouse myometrium [27] and in the cervix [47] during PP period but not during TL. This suggests that cytokine secretion may facilitate the recruitment of immune cells into the adjacent uterine compartments to serve a similar biological function during PP involution.

**Fig. 5:**
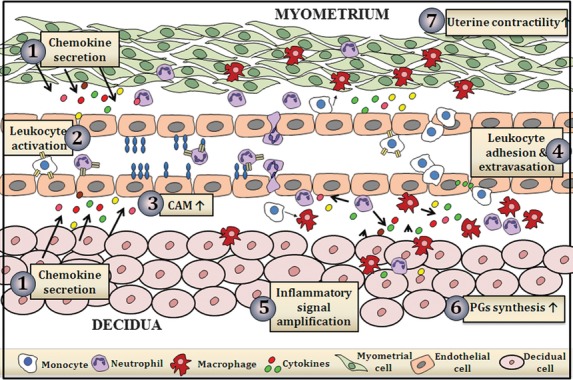
Schematic presentation of the hypothetical model of labour initiation. (**1**) Uterine chemokines secreted near term (**2**) activate circulating leucocytes and (**3**) cell adhesion molecules (CAMs) on endothelium. (**4**) Peripheral leucocytes adhere to vascular endothelial cells and extravasate into the uterine tissues, differentiate (**5**) producing further cytokines/chemokines which (**6**) induces decidual prostaglandins synthesis and (**7**) increases myometrial contractility that ultimately leads to the onset of labour.

In summary, we propose a hypothetical model describing a synchronized programme of inflammatory events in myometrium and decidua that would be essential for both the labour onset and the PP remodelling of the uterus (Fig. 5). In this model prior to the onset of labour (term or preterm) chemokines (possibly specific for each uterine compartment) are synthesized and released by the myometrium and decidua. These chemokines activate maternal immune cells causing them to target and infiltrate either the decidua [16] or the myometrium [27]. We suggest that first important early event in the activation of the late pregnant decidua is macrophage/monocyte infiltration and/or increased macrophage proliferation. The relative abundance of macrophages in mouse decidua and myometrium is consistent with studies of human pregnancy, and highlights the potential importance of uterine leucocytes in the labour cascade. We speculate that within each uterine compartment these immune cells differentiate and induce a physiological inflammation. In the decidua, this might lead to the generation of uterotonic agonists such as prostaglandins [8, 20, 21, 48] that could stimulate the myometrium, while inflammation in the myometrium induces the expression of contraction-associated proteins encoding gap junction proteins, agonist receptors and ion channels [27] which leads to the increase in myometrial contractility. It is likely that decidua plays a co-ordinating role at the maternal–foetal interface by inducing myometrial inflammation [16]. We further suggest that subsequent to these events, additional cytokine release from both uterine compartments might lead to activation of neutrophils in the maternal circulation that target the decidua and myometrium after birth. Our data support the hypothesis of Mitchell and Taggart that human labour may involve an interweaving of the pro-contractile and pro-inflammatory systems which integrates and amplifies uterine contractile activity and initiates preparedness of the immune system for the critical healing/remodelling that occurs within the uterus immediately following birth [49]. Neutrophils contribute to matrix remodelling during PP decidual breakdown and involution of the myometrium, a critical event that completes the reproductive cycle. Appropriate and efficient PP remodelling is necessary to protect the entire reproductive tract from pathogens and to allow subsequent pregnancy [46]. Infiltrating immune cells were reported to play a determinant role in initiating and facilitating endometrial breakdown and shedding during menses [50, 51]. It was shown that this infiltration was tightly regulated by a specific group of chemokines [10] similar to that found in current study. Therefore, we speculate that analogous to endometrial changes during menstruation, immune cells attracted to the labouring uterus by specific chemokines may facilitate the inflammation-like processes in the decidua contributing to the restructuring and angiogenesis required for the re-genesis of the endometrium after delivery. The mechanisms which regulate the temporal expression of specific chemokines by the decidua and myometrium, and how this induces the targeted activation and infiltration of peripheral leucocyte subtypes into these two compartments is the subject of our ongoing investigation. Further studies using rodent models will also establish whether the pharmacologic interventions targeting physiological and pathologic decidual/myometrial inflammation are beneficial for PTL prevention.

## Supporting information

**Figure S1** FACS analyses of immune cells infiltrating mouse decidua during TL and PTL. Decidual suspensions were stained with anti-CD45, anti-Gr1 and anti-Neu 7/4. Live leucocytes were gated on CD45 and further analysed on the Neu7/4 *versus* F4/80 dot plot. Neu7/4^++^ cells were further analysed on the Neu7/4 *versus* Gr1 plot. Monocytes were identified as Neu7/4^++^ and Gr1^low^ to Gr1^+/−^ cells. Neutrophils were defined as Neu7/4^+^, Gr1^++^ population. Macrophages were defined as F4/80^++^ or CD11b^++^, Neu7/4^low^ cells. (A) Monocytes infiltrate the decidua immediately before, during TL and PP; Neutrophils increase in number after TL. (B) Neutrophils are significantly increased during LPS-induced PTL but not during RU486-induced PTL; monocytes are not infiltrating decidua during PTL. Macrophages are the largest population of immune cells present in pregnant mouse decidua. They increased in number by GD18 (term) as compared with GD15 and were elevated during spontaneous TL and PP.

**Figure S2** *In situ* localization of leucocytes in mouse decidua during TL and PTL. Neutrophil (A and C) and macrophage (B and D) infiltration in the decidua during normal gestation and TL, during LPS-induced PTL and during RU486-induced PTL. Neutrophils were identified using anti-Neu7/4 antibody and macrophages—using anti-F4/80 antibody. Leucocytes (arrows) stained dark brown in paraffin-embedded tissue sections that were counterstained a blue with haematoxylin. Representative photomicrographs of (A) neutrophils (Neu7/4) and (B) macrophages (F4/80) in the mouse decidua during gestation and term labour. Immunohistochemical examination was performed on sections of uterus from GD15, GD18, during term labour (TL) and 2–6 hrs post-partum (PP). (C) Neutrophil (Neu7/4) and (D) macrophage (F4/80) infiltration in the decidua during LPS-induced and RU486-induced PTL mouse models. Immunohistochemical examination was performed on sections of uterus from GD 16 (Sham or RU Vehicle), day 15/16 preterm labouring mice (125 LPS PTL or RU486 PTL) and 2–4 hrs PP after preterm delivery induced by either LPS or RU486 (125LPS PP or RU486 PP). The negative controls were performed on GD15 uterine tissue samples: Control 1 represents the lack of immunostaining after incubation of decidual tissue with non-specific rat IgG and Control 2 shows staining with anti-rat secondary antibodies in the absence of primary antibody. Magnification = 200×; scale = 20 μm.
