# Supplementary figures and images for: Infiltration of myeloid cells into decidua is a critical early event in the labour cascade and post-partum uterine remodelling
Source: J Cell Mol Med. 2013 Feb 5;17(2):311–24. doi: 10.1111/jcmm.12012 (PMC3822594; doi:10.1111/jcmm.12012)

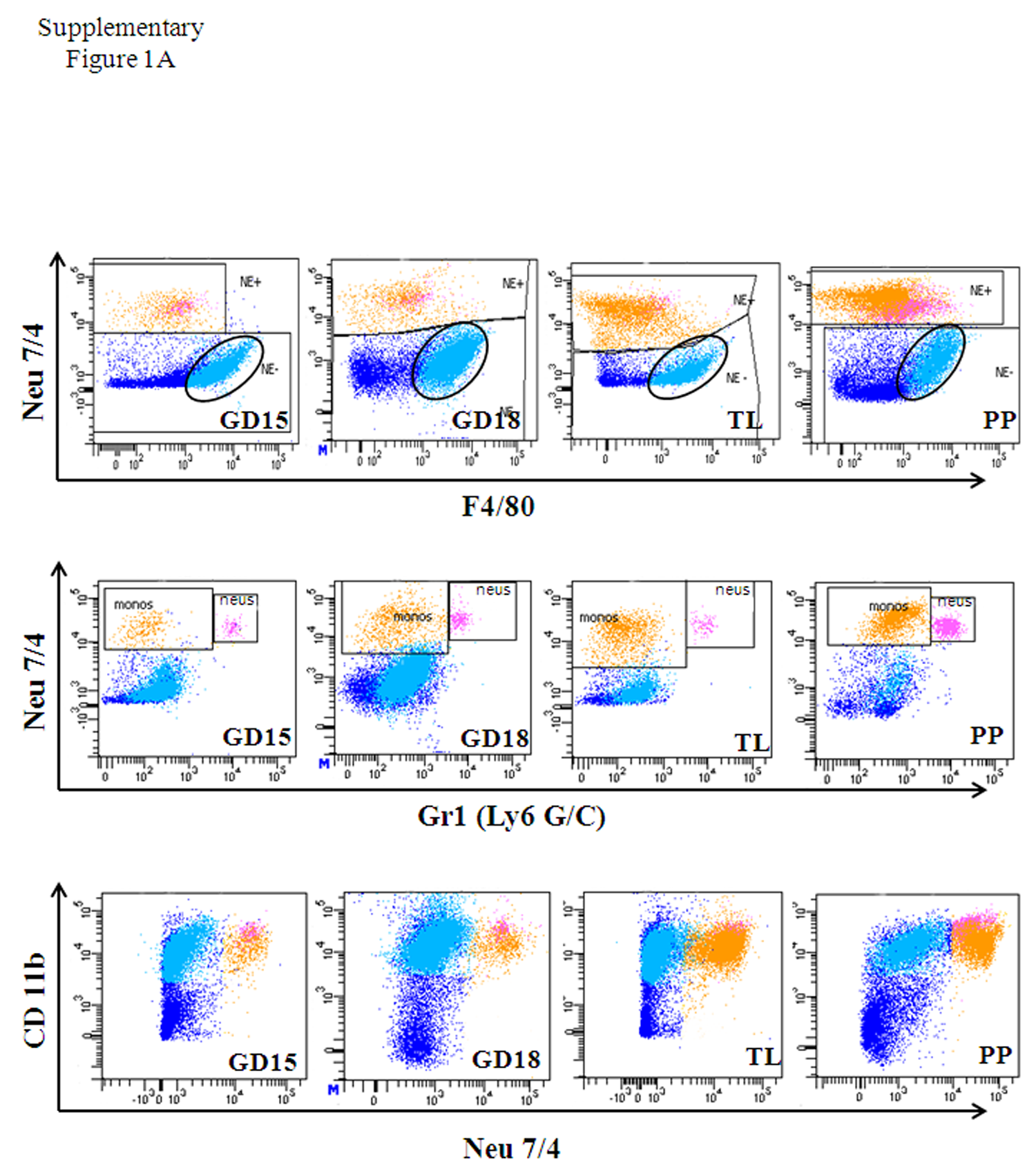

Supplement: Supplementary file 1 [file jcmm0017-0311-SD1.tif]

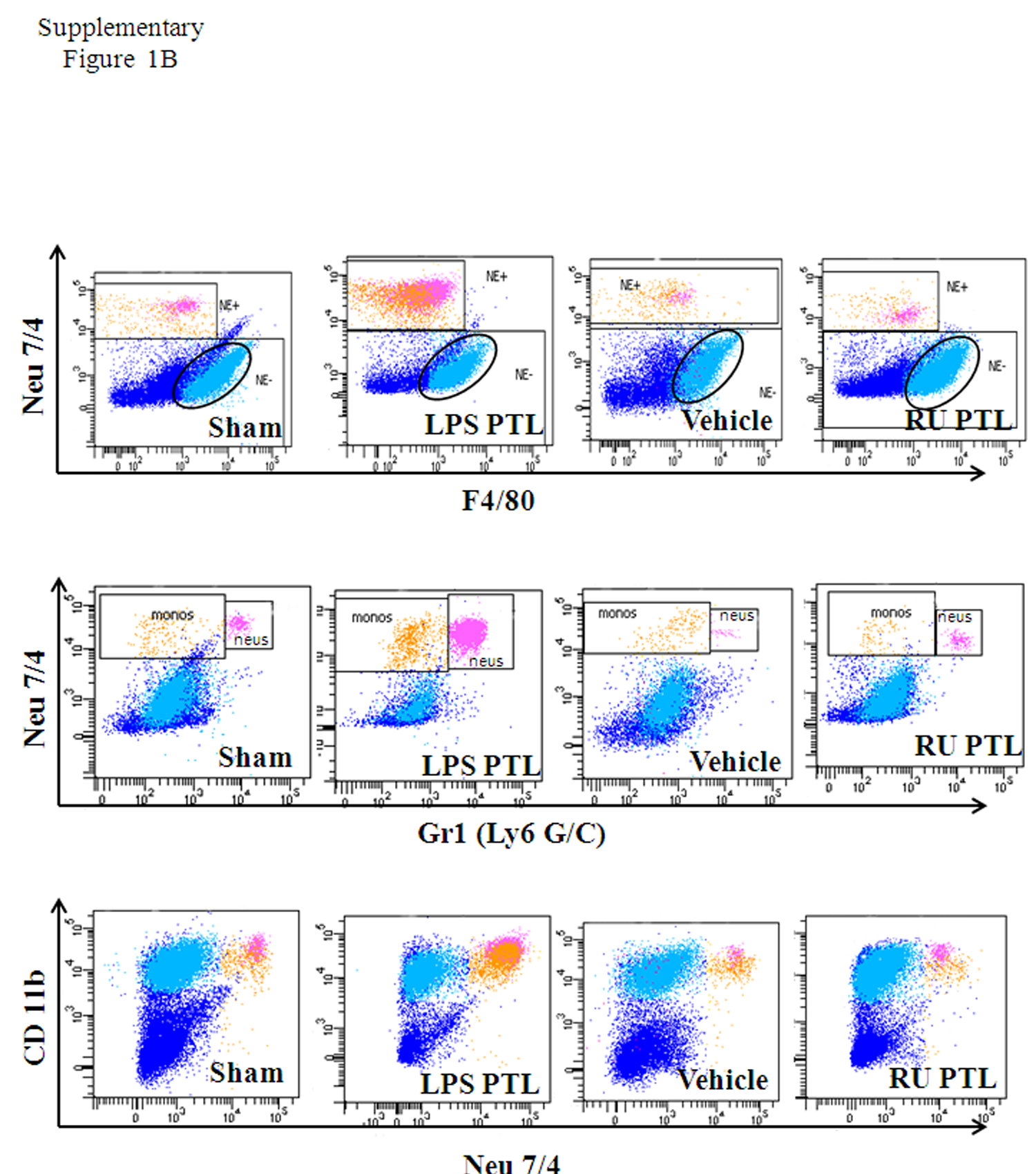

Supplement: Supplementary file 2 [file jcmm0017-0311-SD2.tif]

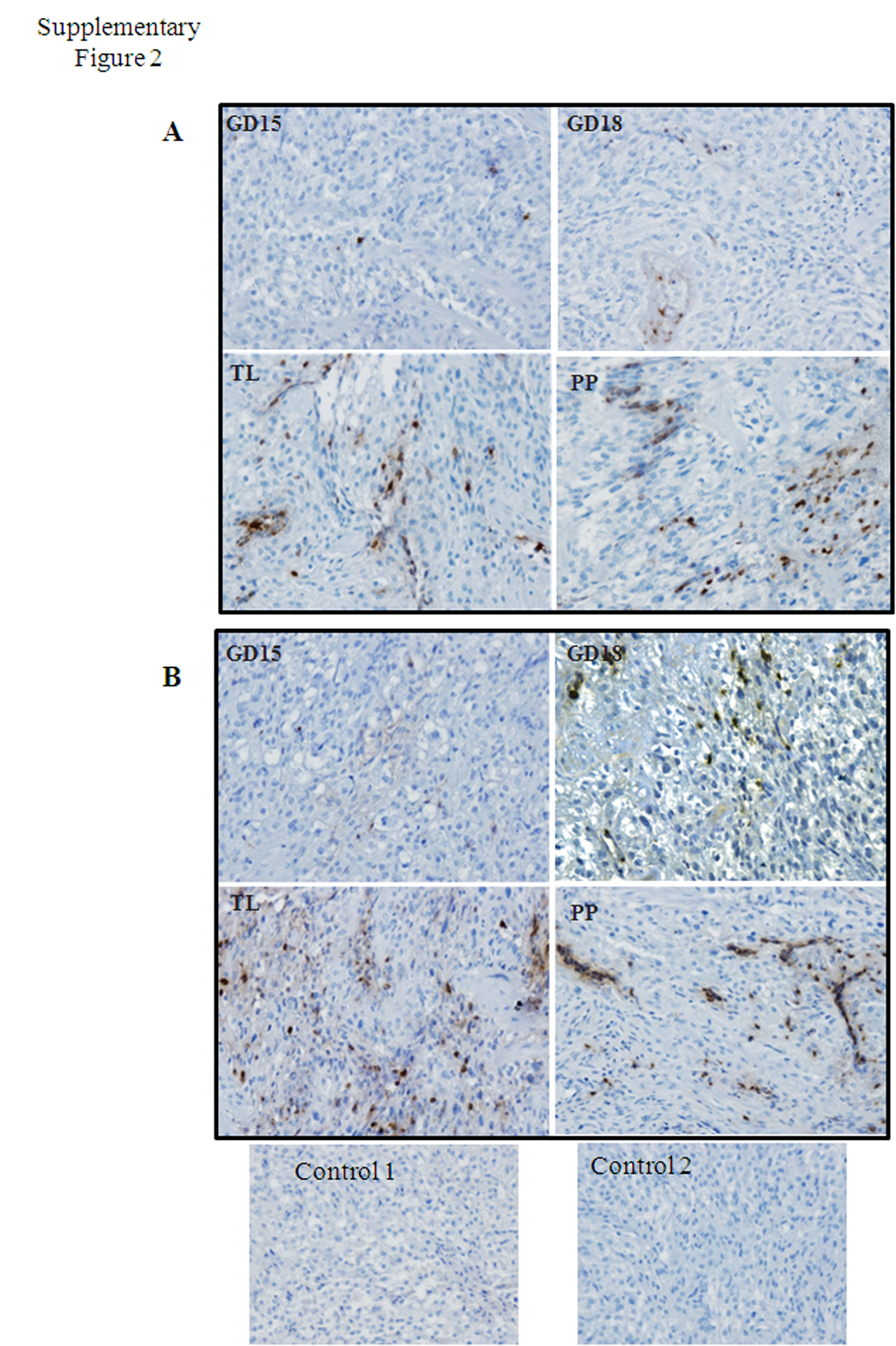

Supplement: Supplementary file 3 [file jcmm0017-0311-SD3.tif]

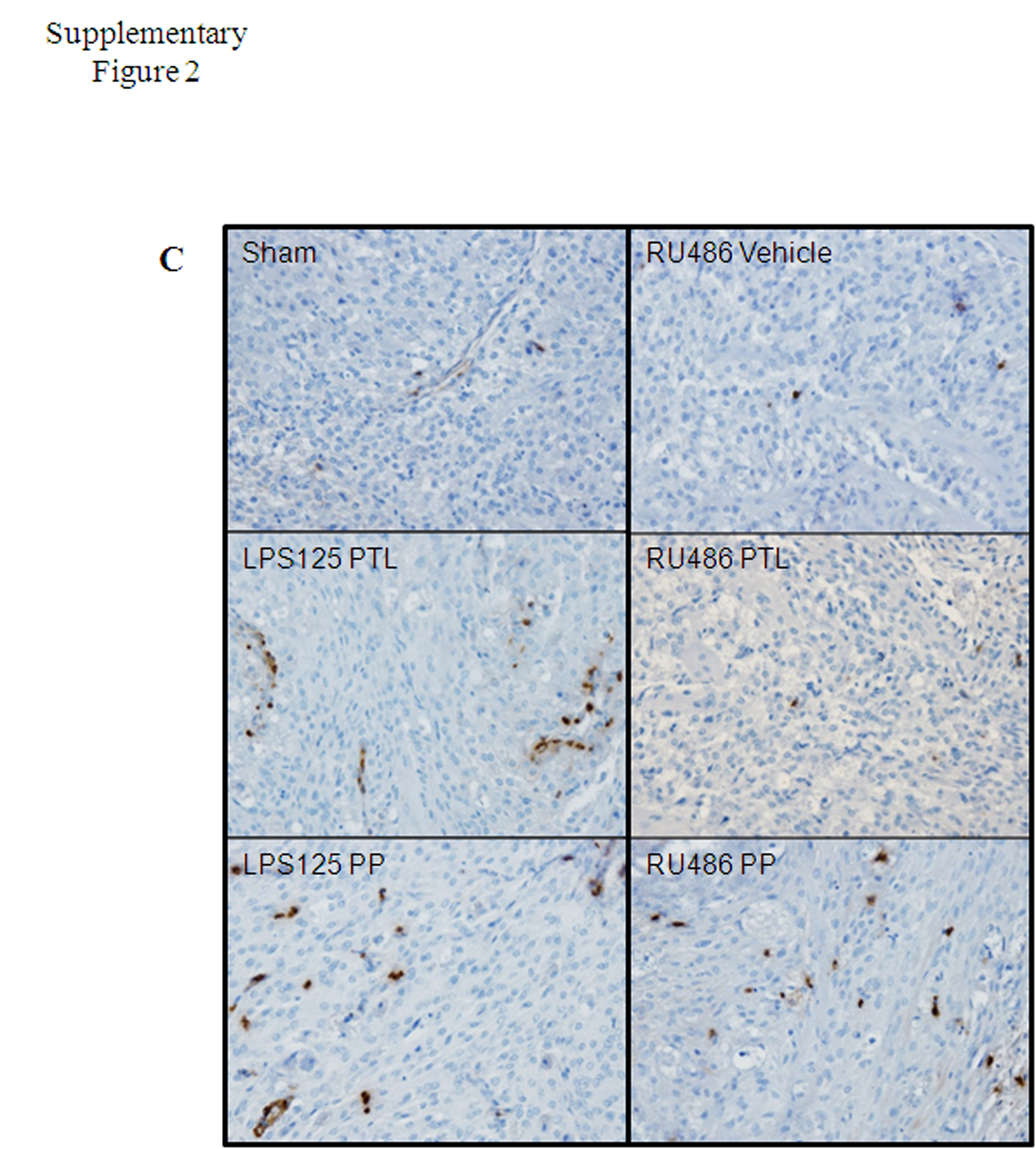

Supplement: Supplementary file 4 [file jcmm0017-0311-SD4.tif]

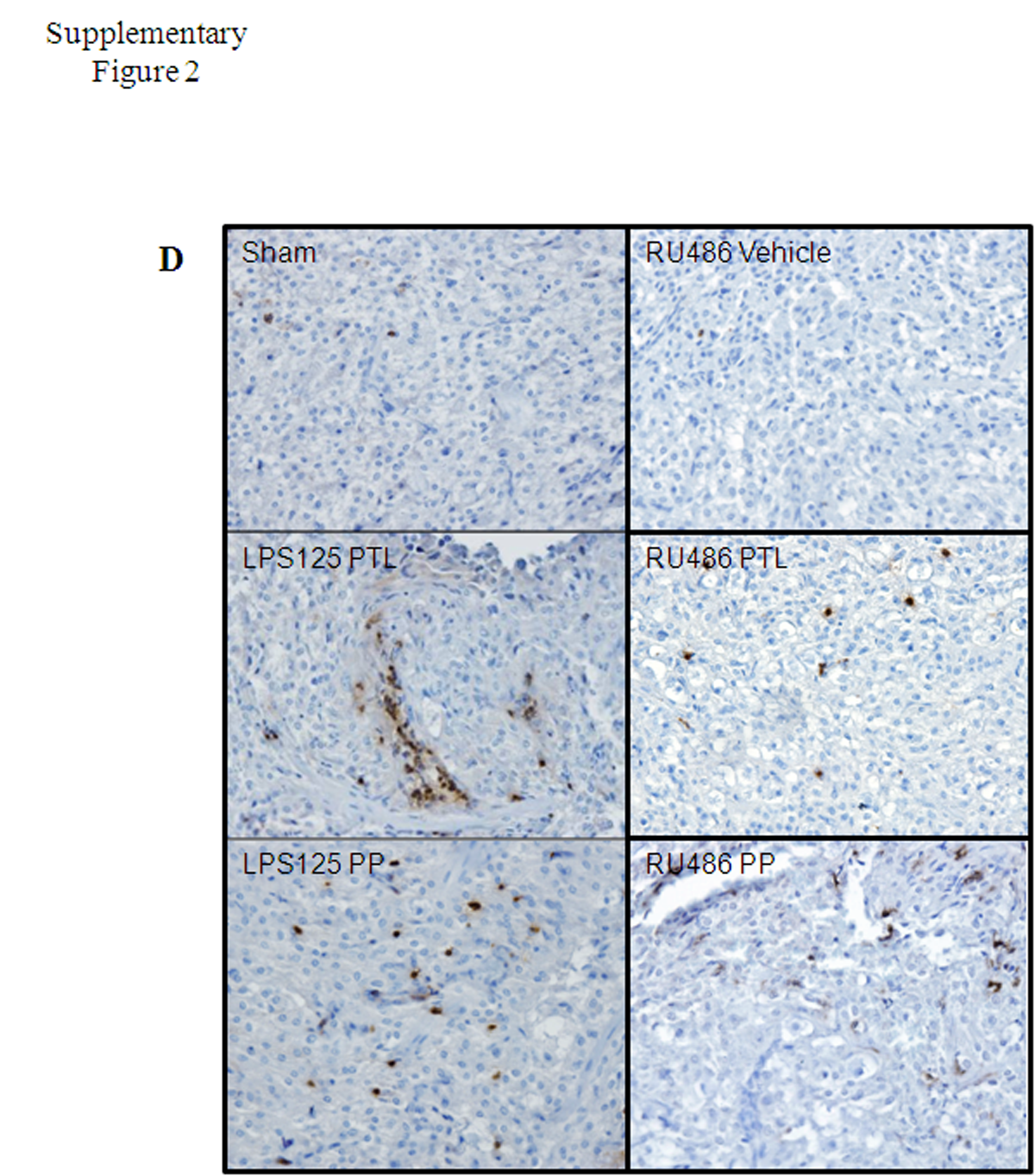

Supplement: Supplementary file 5 [file jcmm0017-0311-SD5.tif]
